# Characterization of the Microstructure and Bonding Properties of Zirconium-Carbon Steel Clad Materials by Explosive Welding

**DOI:** 10.1155/2020/8881898

**Published:** 2020-11-26

**Authors:** Hui Zhao

**Affiliations:** School of Materials Science and Engineering, Xi'an Shiyou University, No.18, Electronic Second Road, Yanta District, Xi'an, Shaanxi, China

## Abstract

An investigation was carried out to characterize the microstructure and bonding properties of the zirconium-carbon steel explosive clad. The microstructure and the composition of the clad were characterized using optical microscopy and scanning electron microscopy. Bonding properties were inspected by using bending and shearing tests. The examination results indicate that the R60702 and Cr70 plates were joined successfully without visible defects. The interface wave is symmetrical. There is no element diffusion across the interface of the clad plate. There are melt blocks at the interface. Bending and shearing test results indicate that the bonding properties of the clad meet the requirements of the ASTM B898 specification. And after shell rolling, no delamination appeared at the interface. Thus, it indicates that the clad plates have good bonding quality and meet the processing requirement.

## 1. Introduction

Zirconium (Zr) and its alloy have excellent corrosion resistance in acid and alkali environment. They show more excellent corrosion resistance than stainless steel, titanium alloy, and nickel alloy. Therefore, Zr is widely used in harsh environments, such as dehydration tower of acetic acid project in the chemical industry, synthesis tower in urea project, and reactor in alcohol production [1–7]. However, Zr is difficult to separate hafnium during refining. Therefore, the price of industrial pure zirconium is relatively expensive, which is 4-5 times that of high-grade stainless steel and 2-3 times that of titanium. Therefore, in recent years, zirconium-steel clad materials are used to replace pure Zr in chemical equipment. The processing method of zirconium-steel clad material is usually explosive welding. In this method, the explosive is used as the energy source, so that the atoms between the two metal interfaces are infinitely close, thus forming a firm combination. However, the physical and chemical properties of zirconium and steel are very different, so it is difficult to clad zirconium and steel by explosive welding method. Thus, Ti is used to the interlayer between zirconium and carbon steel to bond them. The microstructure and properties of zirconium-steel clad materials with Ti interlayer have been investigated by more studies [8–15]. Generally, the interface is the weakness of the clad plate, but the zirconium-steel clad with interlayer has two interfaces. Thus, the study on the zirconium-steel clad materials without the interlayer is useful. However, not too much work has been implemented on zirconium-steel clad materials without the interlayer. Therefore, in this paper, the R60702-Gr70 clad material without the interlayer was prepared by explosive welding. The microstructure and mechanical properties of the clad material were characterized. The results show that the clad material meets the requirements of specification and processing.

## 2. Experimental Procedure

### 2.1. Materials

The flyer and the base plate are zirconium (ASME SB551 R60702) in solution-annealed condition and carbon steel (ASME SA Gr70) in normalization condition, respectively. The chemical compositions of R60702 and Gr70 are listed in Table 1 and Table 2. The flyer and base plate were sequentially prepared with dimensions of 3 × 2330 × 6000 mm and 25 × 2330 × 6000 mm, respectively.

### 2.2. Explosive Welding Process

**Figure 1:**
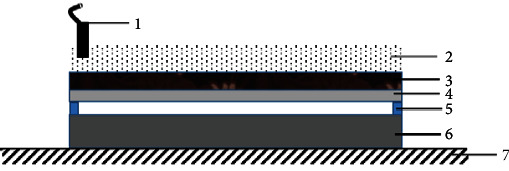
The sketch of explosive welding: (1) detonation, (2) explosives, (3) baffle, (4) R60702, (5) spacer, (6) Gr70, and (7) anvil.

Figure 1 shows the assemble sketch of explosive welding. The R60702 and Gr70 plates were placed in a parallel direction. The spacers (R60702) were put on the surface of Gr70 to support the R60702 plate. The height of the spaces is about 8 mm. The ANFO explosive layer was put on the surface of the R60702 plate. The thickness of the explosive was 33-35 mm, the density was 0.75 g/com^−3^, and the velocity was 2300 m/s^−1^. An electronic detonation was placed in the long edge of the pate. By igniting the electronic detonator, the detonation initiated the explosives, and the R60702 plate was accelerated to shock rapidly the Gr70 plate.

### 2.3. Specimen Characterization

Ultrasonic examination (UT) was employed to inspect the clad plates according to ASTM A578 specification with the Hanwei HS150 equipment [16]. After that, samples were cut parallel to the detonation direction. The cross-sections of specimens were grounded with SiC sandpaper up to No. 2000 and polished. The micrographs of R60702, Gr70, and the interface were examined by optical microscopy (Olympus GX51). The cross-sectional observation of the clad plate after explosive welding and their element analyses were conducted using a scanning electron microscopy (SEM, SSX-550) equipped with an energy dispersed X-ray microanalyzer (EDX). The specimens for the shearing test were prepared according to the ASTM B898[17]. The shearing test was carried out on the testing machine (DLY-10A). The shearing speed was 0.2 mm/min^−1^. The bending test was also performed with the same instrument. The specimens for outside bending were bent up to 105°. And the specimens for inside bending were bent up to 180° (according to specification ASTM B898).

## 3. Results and Discussion

**Figure 2:**
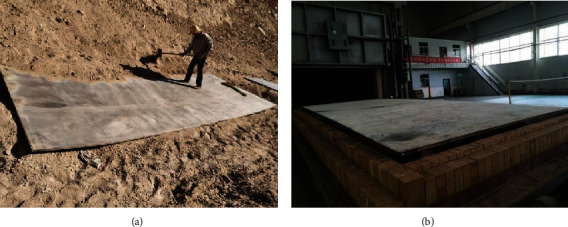
Photographs of the R60702-Gr70 clad plate after (a) explosive welding and (b) flattening (plate size: 3/25 × 2330 × 6000 mm).

**Figure 3:**
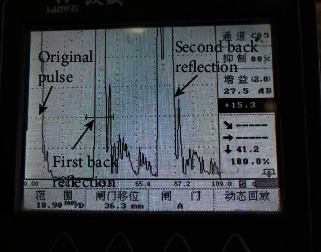
UT record of the clad plate.

Explosive welding on the R60702 plate and Gr70 plate were carried out using ANFO explosives; then, the size of the clad plate was 3/25 × 2330 × 6000 mm.  Figure 2 shows the photographs of the clad plate. During the explosive welding process, the flyer plate impacted drastically to the base plate, leading to the clad plate appeared apparent plastic deformation (Figure 2(a)). Thus, after explosive welding, the clad plate was flattened (Figure 2(b)). As shown in the two pictures, there is no a crack or disjoint defect on the clad plate by visual inspection. For further confirmation of bonding quality, UT was employed to check the continuities of the clad plate. During the UT process, high-frequency ultrasonic energy is introduced and propagated through the R60702 material of the clad plate in the form of pulse. Once there is a defect (such as the unbounded area) in the pulse path, parts of the energy will be reflected back from the defect surface. In the present work, the UT records show that there is no area where one or more discontinuities produced a continuous total loss of back reflection pulse.  Figure 3 is a UT record of the clad plate. There are the first back reflection and the second back reflection pulses in the clad plate. According to the ASTM A578 specification, it indicates the interface of the R60702-Gr70 clad plate is continuous. Thus, the bonding quality of the clad plate meets the requirement of ASTM A578.

**Figure 4:**
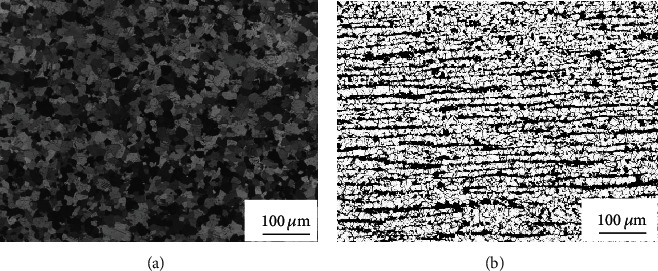
Optical micrographs of (a) R6070 and (b) Gr70.

**Figure 5:**
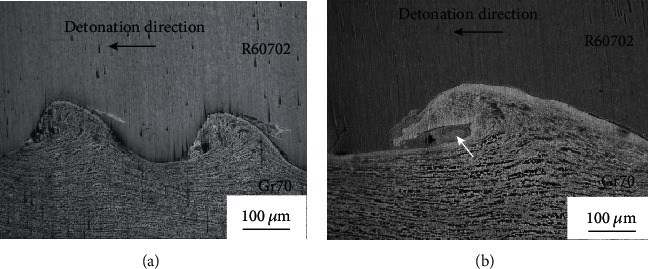
Optical micrograph of the joint cross-section (a) at low magnification and (b) at high magnification.

After UT, microstructure analysis was implemented on the R60702 plate clad, Gr70 plate, and clad plate. Optical views of the microstructures of the clad plate are shown in Figures 4 and 5.  Figure 4(a) shows the microstructure of the R60702 plate. It can be seen from the picture that zirconium alloy (R60702) consists of the *α* phase with a large grain size (about 20-40 *μ*m). The size of *α*-Zr grains is about 8.5 level. The structure is made up of fully equal-axed grains. And there is no abnormal deposition in the metal structure.  Figure 4(b) illustrates the microstructure of Gr70. This steel contains ferrite and pearlite grains with a band-like distribution.  Figure 5 shows the optical interface micrograph of the clad plate. It can be seen from Figure 5(a) the explosive welding process led to the formation of a wavy interface along with the detonation direction. The formation mechanism of this wavy interface is when R60702 and Gr70 plate collided at a high velocity with an oblique angle; a high-velocity cumulative jet was spouted from the squeezed surface of both plates, and behaves as inviscid fluid [18]. The flow pattern of the jet depends on the destiny of the plates being joined [19] The cumulative jet moved along the bisector of the collision angle to form a symmetrical wavy interface in the R60702-Gr70 plate because of their similar densities (shown in Figure 5(a)), as explained in the swinging wake model [20], as shown in [19–21]. And there are no visible defects like pores or cracks in the interface at high magnification (Figure 5(b)). However, during the explosive welding, the R70602 plate collided the Gr70 plate at high velocity (above 1000 m per microsecond); the mechanical energy released during collision leads to a rapid pressure boost, intense plastic deformation in the interface area, friction, and shear of the two materials. This leads to partial melting of the wave crest regions and to the formation of melt blocks (Figure 5(b), white arrow).

**Figure 6:**
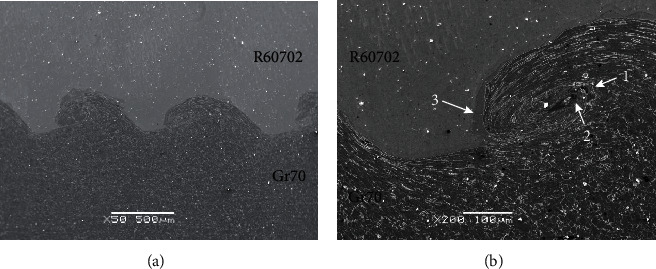
SEM microstructure of the joint cross-section (a) at low magnification and (b) at high magnification.

**Figure 7:**
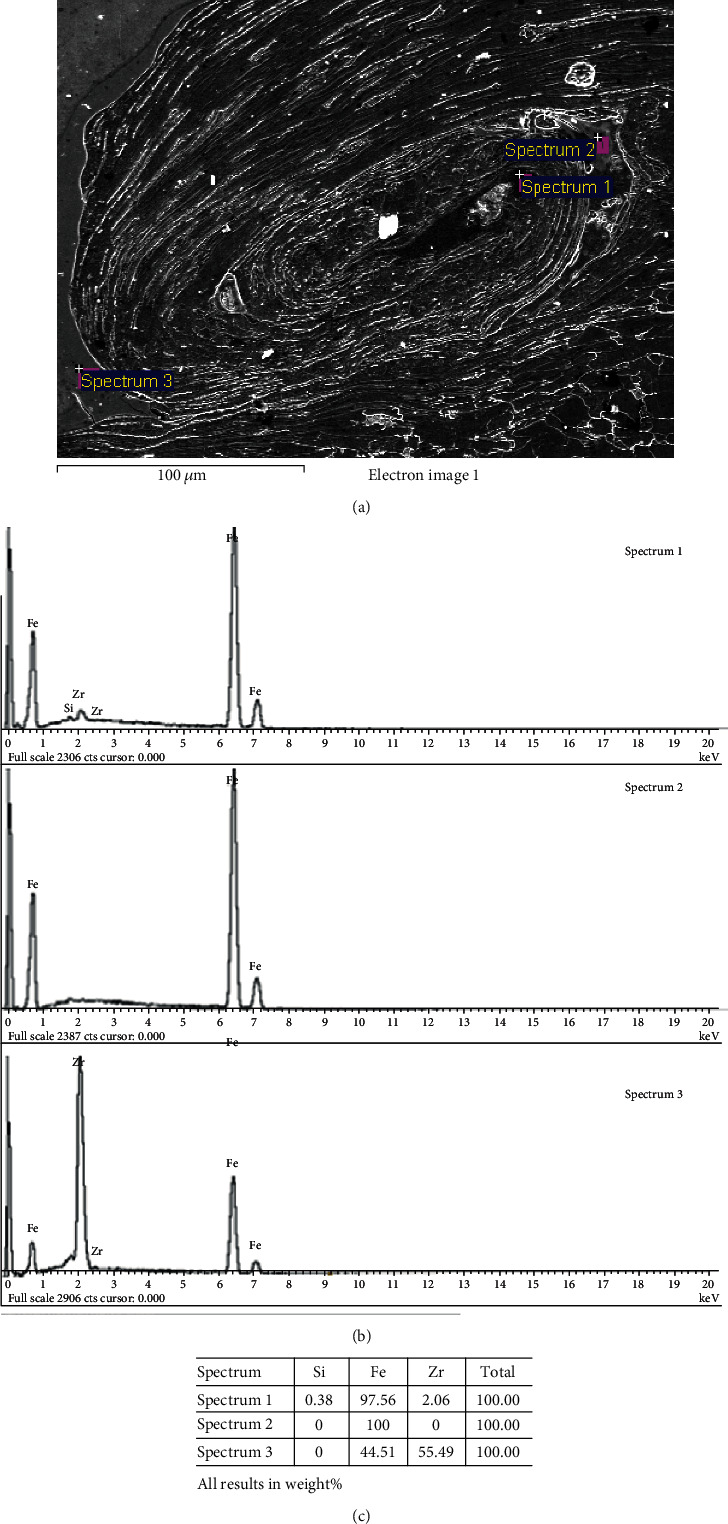
SEM micrographs of the clad plate (a) and corresponding EDX spectrums: (b) white arrow 1 (spectrum1), (c) white arrow 2 (spectrum2), and (d) white arrow 3 (spectrum3).

To further observe the microstructure characteristic of the welded interface, the SEM was employed to investigate the bonding interface as shown in Figure 6(a). And there are also no defects like pores and cracks on the surface of the clad plate. The results are in accordance with that of optical micrographic examination. And base on the results mentioned above, high-velocity collision leading to the temperature increase in local zones, melt blocks formed at the vortices in the clad surface (Figure 6(b)). The elemental components of the melt blocks were measured using EDX at the positions marked with white arrows 1, 2, and 3, in Figure 6(b). The results are shown in Figure 7. The spectrum positions in Figure 7(a) are corresponding to the arrows in Figure 6(b). As confirmed by the EDX analysis, the region at arrow 1 consists of 97.56% Fe, 2.06% Zr, and 0.38% Si; the region at arrow 2 consists of 100% Fe; and the region at arrow 3 consists of 44.51% Fe and 55.49% Zr (Figures 7(b) and 7(c)). Base on the EDX analysis results, it can be concluded that R60702 and Gr70 melted during the explosive welding, and the temperature at the welding interface exceeded the melting temperature of R60700 (1852°C) in a short time. The melt blocks resulted from the melting and mixing of the R60700 and Gr70 plates.

**Figure 8:**
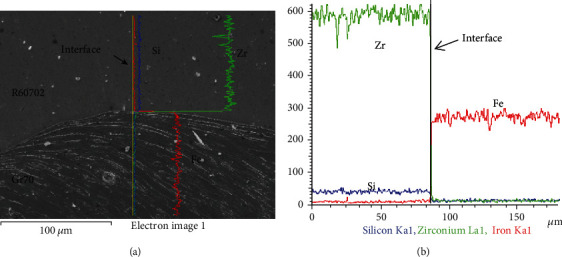
The bonding interface in the R60702-Gr70 clad plate: (a) microstructure; (b) line scan analysis from R60702 to Gr70 plates.

Because of the high temperature at the interface during the explosive welding, it could lead to the formation of the element diffusion across the interface [19–21]. However, Figure 6 indicates that there is no diffusion layer across the interface. In order to confirmation the element diffusion process (possible), line scanning of Fe and Zr, Cr, and Si elements are carried out away from 200*μ*m across the interface. The results are shown in Figure 8. The R60700 and Gr70 materials contain different elements. At the bonding interface from the R60702 to Gr70, it can be seen from Figure 8(b) that the contents of Fe and Zr elements all show sharp transitions across the interface. It indicates that element diffusion during the explosive welding process is not observed under the accuracy of line scanning detection. And there is no silicon in R60702 material (Table 1), thus the silicon element in the specimen is from SiC sandpaper contamination during polishing.

**Figure 9:**
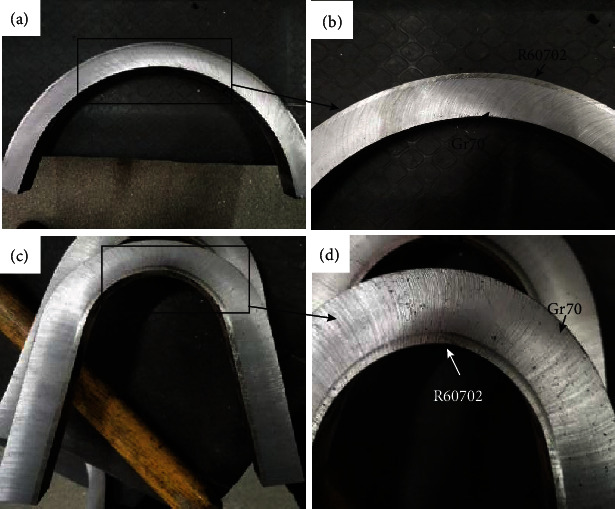
The specimens after bending test: a) and b) outside bending (bended up to 105°), c) and d) inside bending (bended up to 180°).

**Figure 10:**
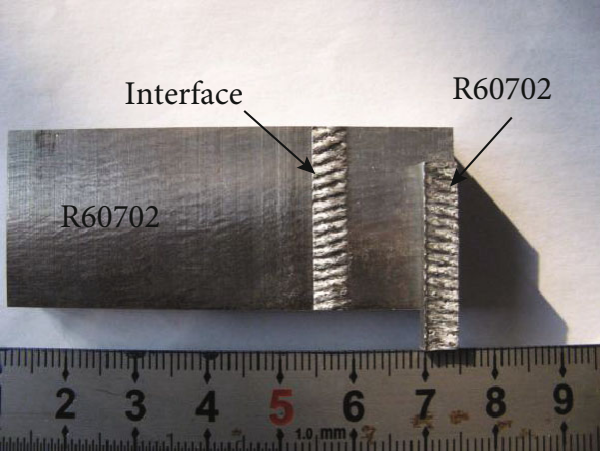
View of the samples after the shearing test.

**Figure 11:**
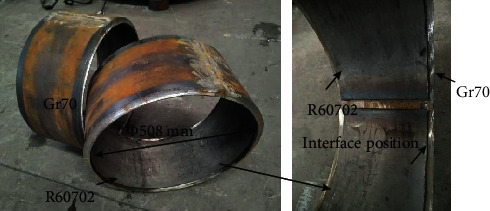
The shell structure after rolling.

Figures 9 and 10 show the mechanical properties of the R60702-Gr70 clad plate. The bending test was conducted to evaluate the bonding quality of the clad plate.  Figure 9 shows the cross-sectional images of the R60702-Gr70 specimen after the bending tests. During the face-bend test, the R60702 plate was subjected to tensile stress, and the Gr70 plate was subjected to compressive stress. During the reversed-bend test, the R60702 plate was subjected to compressive stress, and the Gr70 plate was subjected to stress. Either face-bend specimen or reversed-bend specimen was not delaminated at the interface or parent metals. The result meets the requirements of the ASTM B898 specification. The shearing test was also employed to evaluate the bonding quality of the clad plate. The shearing strength is 412 MPa. It is much higher than that of the ASTM 898 requirement (137 MPa).  Figure 10 shows the shearing specimen after the shearing test. It can be seen that the separation appears at the interface. After the above examination, the clad plate was cut and rolled to the shell structure (shown in Figure 11). The diameter of the shell is *φ*508 mm. During the rolling, the clad plate was not delaminated at the interface or base metals. It indicates the clad plate meets the processing requirement.

## 4. Conclusion

An investigation on the R60702-Gr70 explosive clad plate was carried out to understand the microstructure and bonding properties. The important findings are as follows:

1. The R60702-Cr70 clad plate with 3/25 × 2330 × 6000 mm was successfully obtained by the explosive welding technique. UT inspection indicates that the bonding quality meets the requirement of the ASTM A578 specification
2. The R60702-Gr70 explosive clad plate has a wavy interface. The wave is symmetrical. No cracks or pore defects were observed at the interface. And there is no element diffusion across the interface of the clad plate
3. There are melt blocks on the interface. These melt blocks resulted from the melting and mixing of the R60702 and Gr70 materials during the high-velocity collision
4. Bending test results indicate that no delamination appears at the interface. And the shearing strength reaches 412 MPa. All the results meet the requirements of the ASTM B898 specification. Also, after shell rolling, there is no delamination that appears at the interface. It indicates that the clad plate has good bonding quality and meets the processing requirement

## Figures and Tables

**Table 1:** The components of R60702.

Material	Chemical element (%)						
	Hf	Fe+C	C	N	H	O	Zr
ASTM SB551 R60702	≤4.5	≤0.1	≤0.05	≤0.025	≤0.005	≤0.10	Bal.
Measured values	2.21	0.066	0.007	0.003	0.001	0.068	Bal.

**Table 2:** The components of Gr70.

Material	Chemical element (%)					
	C	Si	Mn	P	S	Fe
SA 516 Gr70	≤0.20	≤0.035	1.20~1.60	≤0.015	≤0.015	Bal.
Measured values	0.18	0.015	1.14	0.005	0.008	Bal.

## Data Availability

I declare that I do not have any commercial or associative interest that represents a conflict of interest in connection with the work submitted.

## References

[1] Havenga J. L., Nel J. T. The Manufacturing of Zirconium Metal Powder by Means of a High Temperature Plasma Process.

[2] Li Y., He J. (2018). Comparative and verified studies of zirconium nanocomposite nanofibres by bubble spinning. *Micro & Nano Letters*.

[3] Gayvoronsky I. V., Girzhon V. V. (2015). Structural state of zirconium surface layers after laser alloying by titanium and nickel. *2015 International Young Scientists Forum on Applied Physics (YSF), Dnipropetrovsk*.

[4] Pushilina N. S., Kudiiarov V. N., Nikolaeva A. N. (2014). Investigation of hydrogen distribution in zirconium alloy by means glow discharge optical emission spectroscopy. *2014 9^th^ International Forum on Strategic Technology (IFOST)*.

[5] Kaushik S., Buddhadev K., Sangita D. (2020). Direct non-destructive total reflection X-ray fluorescence elemental determinations in zirconium alloy samples. *Journal of Synchrotron Radiation*.

[6] Yang L. L., Chen M. H., Wang J. L. (2020). Microstructure and composition evolution of a single-crystal superalloy caused by elements interdiffusion with an overlay NiCrAlY coating on oxidation. *Journal of Materials Science & Technology*.

[7] Qiao Y. X., Huang J., Huang D. (2020). Effects of laser scanning speed on microstructure, microhardness, and corrosion behavior of laser cladding Ni45 coatings. *Journal of Chemistry*.

[8] Aushev A., Balandina A. N., Grishin E. N., Drennov O. B., Podurets A. M., Tkachenko M. I. (2018). Features of uniaxial and biaxial deformation of the contact boundary of metals during explosion welding. *Inorganic Materials: Applied Research*.

[9] Yang M., Ao H. M., Zhao S., Xu J. F. (2020). Application of colloid water covering on explosive welding of AA1060 foil to Q235 steel plate. *Propellants, Explosives and Pyrotechnics*.

[10] Sun Z. R., Shi C., Xu F., Ke F., Wu X. M. (2020). Detonation process analysis and interface morphology distribution of double vertical explosive welding by SPH 2D/3D numerical simulation and experiment. *Materials & Design*.

[11] Kuz’min E. V., Lysak V. I., Kuz’min S. V., Kerolev M. P. (2019). Effect of parameters of high-velocity collision on the structure and properties of joints upon explosive welding with simultaneous ultrasonication. *The Physics of Metals and Metallography*.

[12] Zhang T. T., Wang W. X., Zhou J. (2018). Investigation of interface bonding mechanism of an explosively welded tri-metal titanium/aluminum/magnesium plate by nanoindentation. *JOM: the journal of the Minerals, Metals & Materials Society*.

[13] Yang S. Y., Bao J. W. (2018). Microstructure and properties of 5083 Al/1060 Al/AZ31 composite plate fabricated by explosive welding. *Journal of Materials Engineering & Performance*.

[14] Loiseau J., Georges W., Frost D. L., Higgins A. J. (2018). The propulsive capability of explosives heavily loaded with inert materials. *Shock Waves*.

[15] Chen P. W., Feng J. R., Zhou Q. (2016). Investigation on the explosive welding of 1100 aluminum alloy and AZ31 magnesium alloy. *Journal of Materials Engineering and Performance*.

[16] (2017). *Standard specification for straight-beam ultrasonic examination of rolled steel plates for special applications*.

[17] (2016). *Standard specification for reactive and refractory metal clad plate*.

[18] Gupta R. C., Kainth G. S. (1990). Swinging wake mechanism for interface wave generation in explosive welding of metals. *Journal of Applied Mechanics*.

[19] Kiselev S. P., Mali V. I. (2012). Numerical and experimental modeling of jet formation during a high-velocity oblique impact of metal plates. *Combustion Explosion & Shock Waves*.

[20] Saravanan S., Raghukandan K. (2013). Thermal kinetics in explosive cladding of dissimilar metals. *Science and Technology of Welding and Joining*.

[21] Onzawa T., Ishi Y. *Fundamental studies on explosive welding, welding research abroad*.

